# Cerebral white matter blood flow and arterial blood pressure in preterm infants

**DOI:** 10.1111/j.1651-2227.2010.01856.x

**Published:** 2010-10

**Authors:** Klaus Børch, Hans C Lou, Gorm Greisen

**Affiliations:** 1 Department of Neonatology, Rigshospitalet and University of CopenhagenCopenhagen, Denmark; 2 Department of Paediatrics, Hvidovre HospitalÅrhus, Denmark; 3 CFIN, University of Åarhus and Institute for preventive Medicine, University of CopenhagenCopenhagen, Denmark

**Keywords:** Autoregulation, Brain, Cerebral blood flow, Haemodynamics

## Abstract

It is generally assumed that one reason why white matter injury is common in preterm infants is the relatively poor vascular supply.

**Aim:**

To examine whether blood flow to the white matter is relatively more reduced at low blood pressure than is blood flow to the brain as a whole.

**Methods:**

Thirteen normoxic preterm infants had blood flow imaging on 16 occasions with single-photon emission computed tomography (SPECT) using 99Tc labelled hexa-methylpropylenamide oxime (HMPAO) as the tracer. Gestational age was 26–32 weeks. Transcutaneous carbon dioxide was between 4.7 and 8.5 kPa and mean arterial blood pressure between 22 and 55 mmHg.

**Results:**

There was no statistically significant direct relation between white matter blood flow percentage and any of the variables. Using non-linear regression, however, assuming a plateau over a certain blood pressure threshold and a positive slope below this threshold, the relation to white matter flow percentage was statistically significant (p = 0.02). The threshold was 29 mmHg (95% confidence limits 26–33).

**Conclusion:**

Our analysis supports the concept of periventricular white matter as selectively vulnerable to ischaemia during episodes of low blood pressure.

Brain injury in preterm infants involves predominantly white matter, and the periventricular zone is at particular risk. Parenchymatous haemorrhage and cystic leucomalacia are most often seen here. Forty years ago, the vascular anatomy was used to explain this by describing periventricular white matter as a vascular end-zone (1) at special risk for ischaemia.

It has been difficult, however, to demonstrate an association between white matter injury and ischaemia or low cerebral blood flow. Cerebral blood flow (CBF) is moderately low before major intracranial haemorrhage (2,3), and low CBF is weakly associated with neurodevelopmental deficit (4,5). One problem in these studies is that measurement of CBF was random in time. As CBF is likely to vary significantly with pCO_2_ and perhaps also with blood pressure, it is not likely that the minimal value was recorded.

Another problem is that if blood flow to the white matter is selectively decreased, this will not be easy to detect with a measure of global CBF as is obtained by xenon clearance or near infrared spectroscopy.

To address this issue, we used a purpose-built, mobile single-photon emission computerized tomograph to image blood flow in ill newborn infants. We have reported that flow to white matter is extraordinarily low in the human preterm neonate (6).

We have until now only reported our findings in hypotension in the form of a congress abstract (7). This was because of the limited sample size and the scatter of values. The scanner is no longer available. But to our knowledge, no better data have appeared in the literature. That is why we now publish the study in full. We used a post hoc hypothesis for analysis.

## Patients and methods

### Patients

Infants of <33 weeks were enrolled in the study. Exclusion criteria for this analysis were hypoxia (pulseoximeter reading below 88%) and abnormal brain ultrasound before or after the study. It was possible to perform 16 measurements of regional cerebral blood flow (rCBF) in 13 infants fulfilling these criteria. The limiting factors were the clinical condition of the infant, parental consent, and availability of primary investigator (KB), scanner, and tracer. The measurements were performed from March 1992 to March 1994.

### Tracer

^99m^Tc-Labelled HMPAO was prepared by adding 80 MBq of freshly eluted ^99m^Tc in 10 mL of isotonic saline into one vial of HMPAO (Ceretec, Amersham International). The tracer was injected intravenously in a dose of 4 MBq/kg body weight within 30 min from preparation. HMPAO acts as a ‘chemical microsphere’; therefore, imaging can be carried out several hours after an injection of tracer that was made during an acute episode. Imaging could then be carried out when the baby had been stabilized.

### SPECT equipment and procedure

SPECT measures the activity and the position of a radioactive tracer emitting one photon per decay. The activity is proportional to the number of decays with the corrections mentioned below. The position of the tracer is determined by the measurements from different angles. We used a brain dedicated fast-rotating four-head multidetector system (Tomomatic 248®, Medimatic, Denmark). The scanner was mobile, which allowed bedside studies (8).

**Figure 1:**
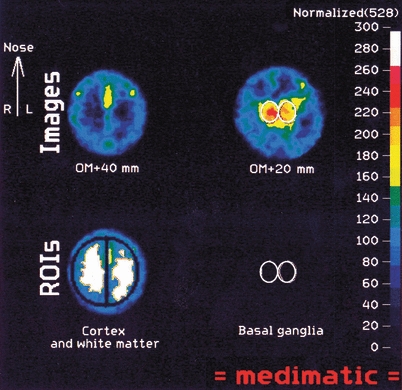
Upper row. Two imaging ‘slices’ 20 and 40 mm above the ear opening. The upper slice images the hemispheric cortex and the centrum semiovale. The lower slice images the basal ganglia/thalamus in the centre. Lower row: The regions of interest: lateral and mesial cortex, white matter, and basal ganglia/thalamus.

The images were reconstructed into 1.25-mm pixels using a filtered back-projection algorithm using a Shepp-Logan filter, and an attenuation correction coefficient of 0.15/cm. The images were normalized to the mean of all slices. The tracer was fixed in the brain within 1 min, but a small part of the tracer diffused back from brain to blood. This back-diffusion is most predominant in the high-flow areas. A correction for this back diffusion of tracer from brain to blood (linearization) was performed using a correction factor of 3.4 (Ref. 2). Image analysis: The images were analysed by a semi-automated method discriminating flow to the cortical grey matter, the basal ganglia and to the white matter (Fig. 1). (Ref. 2). For this analysis the flow to the white matter was expressed as a percentage of the blood flow to the entire brain and labelled WM%.

Informed consent was obtained from the parents of each infant. The local ethics committee of Copenhagen approved the study protocol. The maximal allowed accumulated radiation dose was 5 mSv (effective dose). In reality, average absorbed dose for one scan was 1.5 mSv, and as no infants had more than two studies carried out, maximum radiation dose was 3 mSv.

### Statistical analysis

White matter flow percentage was apparently normally distributed with low skewness and normal kurtosis. Pearson correlation was used first. Then non-linear regression of white matter flow percentage (WM%) vs. mean arterial blood pressure (MABP) was made. The non-linear equation was VM% = (*MABP < threshold*) *(MABP−threshold) * slope+ plateau. This fits a break in the regression line: sloping at low ABP until a threshold and being horizontal at ABP above the threshold. A constraint of the non-linear regression procedure was set not allowing *threshold* to move outside the range 25–35. We used PASW 18.0 (SPSS inc, Chicago, IL, USA).

## Results

Mean birthweight was 986 g (range 550–2680) and the mean gestational age was 27.3 weeks (range 26–32) (Table 1). Mean arterial pressure ranging from 22 to 55 mmHg. Transcutaneous pCO_2_ varied between 4.7 and 8.5 kPa (TINA, Radiometer, Denmark). Nine infants were healthy at 1 year of age and four died due to bronchopulmonary dysplasia without sign of brain damage.

**Table 1:** Clinical and cerebral perfusion data from 16 measurements in 13 infants

												Relative perfusion (%)		
												
Pt. no.	Birthweight (g)	Gestational age (weeks)	Sex	Age at examination (days)	Postmenstrual age at examination (weeks)	Ventilation*	pCO_2_ (kPa)	pO_2_ (kPa)	Saturation (%)	MAP (mmHg)	Abnormal outcome†	White matter	Cortex	Basal ganglia
1	2680	32	Boy	2	32	V	4.70			37		61	112.5	246.5
2	820	26	Girl	1	26	V	5.80	11.70	94	31		60.5	138.5	229
3	730	26	Girl	2	26	NC	6.80	17.20	97	42		51.5	112	275
4	860	27	Boy	1	27	NC	6.00	9.10		26		52	104.5	
				5	28	V	7.20	7.20		48		53.5	101	243.5
5	550	26	Boy	24	29	V	8.50	5.80	92	50	Died	70	104	232
6	860	26	Boy	1	26	NC	5.10	11.30		30	Died	59	100.5	176
7	715	26	Girl	2	26	V	6.00	10.70	95	29	Died	64.5	104.5	221
				21	29	V	6.10	8.20	90	46		48	101	185.5
8	1208	28	Boy	1	28	V	5.90	8.70	92	22		44.5	97	166
				9	29	NC	6.40	5.50	95	55		60	99.5	219
9	800	26	Girl	1	26	V	5.10	8.90	97	25		38	110.5	
10	845	30	Girl	1	30	V	4.90	7.80	89	28		57	117.5	306.5
11	845	30	Girl	10	31	NC	6.20	10.70	95	42		46.5	95.5	191
12	1120	27	Boy	1	27	V	5.50	7.60		29	Died	55	122	205.5
13	955	26	Boy	14	28	NC	6.00	7.50	92	48		71	107.5	228.5

* V = Mechanical ventilation; NC = Nasal CPAP.

† If nothing is stated, the infant was healthy at 1 year of age.

The relative blood flow to white matter did not correlate to gestational age (p = 0.8), birth weight (p = 0.8) or transcutaneous pCO_2_ (p = 0.3). Therefore, correction for pCO_2_ was not attempted. WM% was not simply correlated to MABP either (p = 0.12).

**Figure 2:**
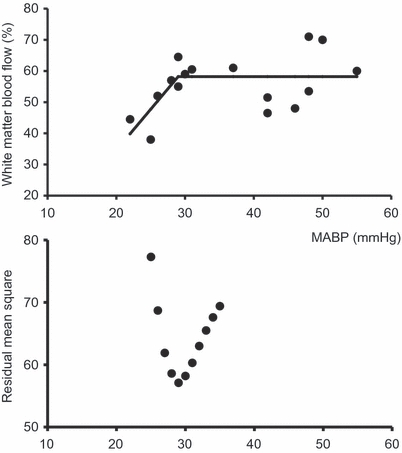
Upper panel: Flow to cerebral white matter – expressed as percentage of global cerebral blood flow as function of mean arterial blood pressure. The 16 measurements were obtained in 13 infants. A continuous function was fitted as a bi-linear regression with a preset breakpoint. Lower panel: The lowest residual mean square (best fit) was obtained with the breakpoint at 29 mmHg.

The non-linear relation between WM% and MABP, in a shape like the conventional concept of autoregulation, was statistically significant (multiple r^2^ = 0.344, 13 df, p = 0.02) with the breakpoint at 29 ± 2.0 mmHg (95% confidence limits 24–34 mmHg), a slope below the breakpoint of 2.6 ± 1.3% per mmHg, and a plateau above the breakpoint of 58.2 ± 2.5% (Fig. 2).

## Discussion

We found support for the concept of the white matter as a vascular end zone. Blood pressure below 29 mmHg was associated with smaller share of blood flow to white matter. The implication is that white matter is selectively vulnerable to ischaemia when blood pressure is low. It also means that measures of CBF that include grey as well as white matter are less sensitive to this and therefore that studies reporting absence of a lower threshold for the autoregulatory plateau in preterm infants (9,10) should be interpreted in this light.

The method used here, HMPAO imaging, is based on the bolus distribution principle. This principle states that the concentration of tracer fixed in tissue after a bolus injection is proportional to the rate of perfusion, that is proportional to blood flow per unit of tissue volume. The uptake of HMPAO during the first pass is less than complete, and more so at higher rates of flow. This problem was compensated for by linearisation. Furthermore, images were not converted to units of flow. This would have required determination of the arterial input that is difficult for HMPAO. We circumvented this by expressing flow to the white matter in relative terms. Thus, it is was not possible to examine the threshold of autorelation in white matter directly, but only to examine whether blood flow to white matter is relatively more reduced at low blood pressure than is blood flow to the brain as a whole. That is why we interpret the result to indicate the white matter is selectively vulnerable to ischaemia during hypotension.

The statistical significance of the result, however, depends on the assumption of a non-linear relation between blood pressure and relative white matter flow of the specified shape. For the analysis we assumed the existence of a plateau and a threshold. The statistical analysis resulted in a stable estimate of the threshold that was in good agreement with expectations. Incidentally, a MABP of 30 mmHg has been used as a practical definition of hypotension in preterm infants based on early observations of risk of cerebral haemorrhage (11), and this was also the threshold for autoregulation found in extremely preterm infants treated with dopamine to support blood pressure (12).

Our study has a number of weaknesses. First, because of the highly technically demanding method, the group of infants was small and was not sampled systematically. Second, in 1992–1994, arterial hypotension was treated more actively than now. This meant that we have only a few studies during significant hypotension. Third, whereas the relation between blood pressure and WM% was statistically significant, two-thirds of the variance of WM% was still unexplained. Finally, two imaging studies were achieved in only three of the 13 infants, precluding any meaningful paired statistical analysis.

The result should not be used for clinical decision-making. At least it should be realised that the decrease in relative flow to white matter at pressures below 30 mmHg was gradual, being only moderate at the pressures recorded. It should also be realised that normal CBF includes a comfortable reserve (13). Measures of cerebral oxygenation with near-infrared spectroscopy in preterm infants suggest that it is also the case for term and preterm infants in general (14,15). In situations where blood flow to cerebral white matter is already marginal, however, such as in cases of low pCO_2_, low blood glucose, or low blood haemoglobin concentration, a low blood pressure may be detrimental.

In conclusion, our data support the concept of periventricular white matter in preterm infants as a vascular end-zone, particularly vulnerable to ischaemia.
